# WHO Establishes New Rapid-Response Unit for Emerging Infectious Diseases

**DOI:** 10.3201/eid0201.960114

**Published:** 1996

**Authors:** Philippe Stroot

**Affiliations:** World Health Organization, Geneva, Switzerland


### References

1. Allworth T, O'Sullivan J, Selvey L, Sheridan J. Equine morbillivirus in Queensland. Communicable Diseases Intelligence 1995;19:575.
2. Murray K, Rogers R, Selvey L, Selleck P, Hyatt A, Gould A, et al. A novel morbillivirus pneumonia of horses and its transmission to humans. Emerging Infectious Diseases 1995;1:31-3.

**Sandra Lane**
Case Western Reserve University
Cleveland, Ohio, USA
E-mail: sxl45@po.cwru.edu

### Social Science and the Study of Emerging Infectious Diseases

Topics related to emerging and reemerging infectious diseases attracted a considerable audience at the annual meeting of the American Anthropological Association, November 15–19, 1995, in Washington, D.C. The meeting had a separate session entitled "Emerging and Reemerging Infectious Diseases: Biocultural and Sociocultural Approaches."

The session brought together anthropologists interested in and working on emerging infectious diseases from various subdisciplinary perspectives. Presentations were made on the following subjects: outline of a research agenda, deforestation and the emergence of infectious diseases in the rain forests of Papua-New Guinea, the cholera epidemic in Latin America, evolutionary aspects of emergent infections, societal impacts of the test for acquired immunodeficiency syndrome, compliance and iatrogenesis in tuberculosis treatment in the United States, patchwork policies that affect long-term treatment of tuberculosis in Nepal and Uganda, the reemergence of schistosomiasis in Egypt, dengue control in Latin America, cultural and political ecologic models of emergent infections, and the politics of leprosy eradication. Abstracts are available from the conference organizers, listed below.

Anthropologists interested in international health and the social science aspects of infectious diseases are organized in a working group called the International Health and Infectious Disease Study Group of the Society of Medical Anthropology (American Anthropological Association). Requests to subscribe to this group's newsletter can be sent to

**Johannes Sommerfeld**
Harvard Institute for International Development
Cambridge, Massachusetts, USA
Phone: 617-495-9791
E-mail: jsommerf@hiid.harvard.edu

### WHO Establishes New Rapid-Response Unit for Emerging Infectious Diseases

The World Health Organization (WHO) has established a new rapid-response unit to control and prevent the growing incidence of new and reemerging diseases worldwide. The unit's focus will be improved containment of disease outbreaks, such as that caused by the deadly Ebola virus, which struck Zaire in 1995.

The WHO unit will be called the Division of Emerging Viral and Bacterial Diseases Surveillance and Control (EMC). It will be capable of mobilizing staff from WHO headquarters in Geneva and from the organization's regional offices.

In addition to mobilizing WHO's own technical staff and expertise, EMC will coordinate the activities of the agency's traditional partners, for example, its international network of collaborating centers, bilateral donors, expert advisers, and nongovernmental organizations.

Teams equipped to implement epidemic control measures will be placed on-site within 24 hours' notification of an outbreak. This strategy, when implemented in Zaire, not only rapidly contained the recent Ebola outbreak but also prevented its spread to Kinshasa, the capital city of 2 million.

Among EMC's goals are 1) to strengthen local surveillance and disease control so that countries can develop the early warning systems needed to detect emerging or reemerging diseases through innovative field epidemiology and public health laboratory training programs and 2) to continue WHO's activities in developing a network of public health laboratories to strengthen regional and international collaboration in outbreak detection and control.

EMC will continue to expand WHO's network—termed WHONET—that detects and monitors antibiotic resistance worldwide. WHO will use the information collected to continue to advocate research and development of new antibiotics to replace those that are no longer effective.

For further information on WHO's rapid-response unit, contact

**Philippe Stroot**
Health Communications and Public Relations
World Health Organization
Geneva, Switzerland
Phone: 41-22-791-2535
Fax: 41-22-791-4858

### Rotavirus Vaccine Workshop Held

More than 125 participants from at least 15 countries attended the Fifth Rotavirus Vaccine Workshop at the Centers for Disease Control and Prevention (CDC) in Atlanta, Georgia, October 16-17, 1995.

Rotavirus has emerged as the most important cause of severe diarrhea in children worldwide. It is a problem not only in developing countries, where it kills an estimated 870,000 children each year, but also in the United States, where it remains the most important single cause of hospitalization or clinic visits for childhood diarrhea.

Moreover, although studies from many countries indicate that only four serotypes are predominant worldwide, some strains at every site studied cannot be serotyped. In some countries such as India, the diversity of strains is extensive. Further studies are needed to define the extent of cross-protection against these strains that is induced by the vaccine to determine whether additional antigens need to be included in vaccines for such areas.

This workshop included sessions on epidemiology, virology, pathogenesis and immunity, and vaccines currently being tested. Each session had numerous presentations by leaders in the field of rotavirus research. Researchers reported that several live oral rotavirus vaccines, based on animal strains of rotavirus combined with reassortant strains, have been tested in field trials in children. These appear to protect American children against rotavirus and are more efficacious against severe disease. These vaccines like natural protection, are not 100% protective so many investigators are exploring alternative approaches to vaccines such as the use of virus-like particles, native DNA, and microencapsulation of antigens.

No published volume of proceedings from the workshop is planned, but a supplemental issue of the *Journal of Infectious Diseases* scheduled for early 1996 will contain papers from the meeting.

The workshop was held under the auspices of the National Institutes of Health, Emory University School of Medicine, and the World Health Organization.

For further information, contact

**Roger I. Glass**
Global Program for Vaccines and Immunizations
World Health Organization
Geneva, Switzerland
Phone: 41-22-791-2698/2681
Fax: 41-22-791-4860

### International Conference Addresses Preparedness for Emerging Strains of Pandemic Influenza

An international meeting on pertinent issues related to recognizing, identifying, and controlling newly emerging strains of pandemic influenza was held in Bethesda, Maryland, December 11–13, 1995. The conference, "Pandemic Influenza: Confronting a Reemergent Threat," was sponsored by the National Institutes of Health, the University of Michigan, the Centers for Disease Control and Prevention, the Food and Drug Administration, the U.S.-Japan Cooperative Medical Science Program, and the World Health Organization.

Epidemic strains of influenza cause infections almost every year throughout the world because of continuous minor genetic changes in the virus. However, periodically a major change occurs, such as reassortment between mammalian and avian strains of the virus. These pandemic strains are novel to the human immune system and, therefore, can cause substantial disease worldwide. The conference concentrated on issues that would be crucial to controlling an influenza pandemic.

Plenary and workshop sessions examined the following topics: Can pandemics be predicted? What are the specific approaches for pandemic control? What are the advantages and limitations of vaccines and antiviral agents? The workshops also focused on factors contributing to the emergence of pandemic strains and various aspects of surveillance, such as the adequacy of current global surveillance structure for early identification of a pandemic strain, the use of virologic and

The World Health Organization (WHO) has established a new rapid-response unit to control and prevent the growing incidence of new and reemerging diseases worldwide. The unit's focus will be improved containment of disease outbreaks, such as that caused by the deadly Ebola virus, which struck Zaire in 1995.

The WHO unit will be called the Division of Emerging Viral and Bacterial Diseases Surveillance and Control (EMC). It will be capable of mobilizing staff from WHO headquarters in Geneva and from the organization's regional offices.

In addition to mobilizing WHO's own technical staff and expertise, EMC will coordinate the activities of the agency's traditional partners, for example, its international network of collaborating centers, bilateral donors, expert advisers, and nongovernmental organizations.

Teams equipped to implement epidemic control measures will be placed on-site within 24 hours' notification of an outbreak. This strategy, when implemented in Zaire, not only rapidly contained the recent Ebola outbreak but also prevented its spread to Kinshasa, the capital city of 2 million.

Among EMC's goals are 1) to strengthen local surveillance and disease control so that countries can develop the early warning systems needed to detect emerging or reemerging diseases through innovative field epidemiology and public health laboratory training programs and 2) to continue WHO's activities in developing a network of public health laboratories to strengthen regional and international collaboration in outbreak detection and control.

EMC will continue to expand WHO's network—termed WHONET—that detects and monitors antibiotic resistance worldwide. WHO will use the information collected to continue to advocate research and development of new antibiotics to replace those that are no longer effective.

For further information on WHO's rapid-response unit, contact

Philippe Stroot, Health Communications and Public Relations, World Health Organization, Geneva, Switzerland; Phone: 41-22-791-2535; Fax: 41-22-791-4858
